# Education as a protective factor for mental health risks among youth living in highly dangerous regions in Afghanistan

**DOI:** 10.1186/s13034-022-00548-w

**Published:** 2023-01-23

**Authors:** V. Kovess-Masfety, R. L. Frounfelker, K. Keyes, E. Karam, Ajmal Sabawoon, Bashir Ahmad Sarwari, M. Husky, N. Kaur, C. Rousseau

**Affiliations:** 1 LPPS, University of Paris, Paris, France; 2 Department of Psychiatry, McGill University, Montreal, Canada; 3 Department of Community and Population Health, College of Health, Lehigh University, Bethlehem, PA USA; 4 Mailman School of Public Health, Columbia University, New York, NY USA; 5 Institute for Development, Research, Advocacy & Applied Care (IDRAAC), Beirut, Lebanon; 6 Department of Psychiatry & Clinical Psychology, Faculty of Medicine, St. George Hospital University Medical Center University of Balamand, Beirut, Lebanon; 7 Governance Institute of Afghanistan (GI-A), Kabul, Afghanistan; 8 Kabul University of Medical Sciences (KUMS), Kabul, Afghanistan; 9 Department of Mental Health & Substance Abuse, Primary Health Care Directorate, Ministry of Public Health, Kabul, Afghanistan; 10 Laboratoire de Psychologie EA4139, Université de Bordeaux, Bordeaux, France

**Keywords:** Traumas, Mental health, Children, Education, SDQ, Afghanistan

## Abstract

**Background:**

Children in Afghanistan live in dangerous areas, and have been exposed to traumatic events and chaotic education. Progress has been made on access to education for girls who were the most affected by traditional attitudes against engagement in education.

**Objectives:**

The objectives were to evaluate the mental health of Afghan children living in regions of conflict and the association of mental health with school attendance for girls and boys.

**Method:**

The study included 2707 school aged children in eight regions of Afghanistan (16 provinces) residing in households recruited through a multi-stage stratified cluster sampling strategy in 2017. The level of terrorist threat was evaluated by the intensity of terrorist attacks recorded that year in each province. Child mental health was assessed with the parental report Strengths and Difficulties Questionnaire (SDQ) along with information on school attendance, sociodemographic characteristics and geographic location.

**Results:**

A total of 52.75% of children had scores above threshold for the SDQ total difficulties score, 39.19% for emotional difficulties, 51.98% for conduct challenges, and 15.37% for hyperactivity/inattention. Peer relationship problems were high (82.86%) and 12.38% reported that these problems impacted daily life. The level of terrorist threat was associated with SDQ total difficulties (Adjusted Odds Ratio [AOR] = 4.08, P < 0.0001), with youth in regions with high levels of terrorist threat more likely to have problems than youth in regions with low or medium levels of danger, independent of region and ethnicity. School attendance was negatively associated with emotional symptoms (AOR = 0.65, P < 0.0001) and mental health difficulties with impairment (AOR = 0.67, P = 0.007), but positively associated with peer relationships difficulties (AOR = 1.96, P > 0.0001). Conduct (AOR = 1.66, P < .0001) and SDQ total difficulties (AOR = 1.22, P = 0.019) were higher among boys. Overall, gender did not modify the relationship between school attendance and child mental health.

**Conclusion:**

Attending school is essential for children’s mental health, across gender, and should be supported as a priority in Afghanistan despite the return of the Taliban.

## Background

Afghanistan has suffered from endemic war-related violence, and persistent war has resulted in the destruction of Afghanistan’s economic, social and cultural infrastructures. War and political violence are associated with high rates of mental health disorders, and are associated with the risk of long-term mental health problems [1]*.* Afghan children constitute a particularly vulnerable group for these outcomes. A study on the consequences of armed conflict on children established that, even though most younger children in Afghanistan were not directly exposed to war-related events, such as bombings, physical attacks, or killings, they still suffered from indirect traumatic consequences of the war, such as extreme poverty leading to child labor, together with the psychological consequences of their parents’ deteriorated mental health status [2]. Among Afghan school-attending children, as many as 39% reported exposure to at least one war-related traumatic event in their lifetime, which adds to this group’s high level of exposure to domestic violence (mostly toward their mothers, but also towards them), with a higher prevalence for boys than girls [3].

As with many countries, Afghanistan has a 6–3–3 formal education structure. Primary school has an official entry age of six and seven years and a duration of six grades. Secondary school is divided into two cycles: lower secondary consists of grades 7–9, and upper secondary, also know as high school, consists of grades 10–12. After graduation from high school, there is an entrance exam for entry to bachelor-level education. Education is free from primary to high school for all Afghans, as well as those who succeed in the entrance exam to governmental university. However, in addition to public or governmental institutions, there are private schools and universities that charge fees to students. Educational attainment increases with increasing household wealth among both women and men. In addition, urban children are considerably more likely than rural children to attend both primary and secondary school. In some parts of the country, a shortage of schools and insufficient transportation are the main obstacles to education—a long walk to school means fewer children go. Geographical barriers, especially in mountainous areas, also make it hard for children to reach the classroom. The need to work or earn money was more often cited as a reason for boys never attending school than for girls. The main reasons for males dropping out of school are the need to work and the need to help at home. Among females, 30% dropped out because their parents did not send them to school, while 19% dropped out because they got married [4]*.*

War in Afghanistan has also caused major disruptions in education, but information on the scope of this disruption is limited, especially regarding girls’ access to schools. The Gross Enrollment Ratio (GER)^1^ has increased over time, but improvements in gender parity enrollment have been slow. Due to Taliban rules, most girls’ schools were closed between 1996 and 2001, causing girls’ GER to fall from 32.0% to 6.4%. However, enrollment campaigns, such as the “Back to School” campaign, have increased enrollment numbers among girls in the past two decades. From 2001 to 2013, the total number of schools multiplied by four (3500 to 14,600) [5]. However, boys and girls either attend separate schools or separate shifts in the same schools. By 2015, female enrollment in primary school represented 56% of the male rate, and this rate dropped further in secondary school [6]. Gender disparities in education enrollment are particularly severe in rural areas where schools are scarce and difficult to access. Afghan parents tend to prefer female teachers and single-gender classrooms for their daughters, but there is a shortage of all-girl schools and female teachers, which negatively affects girls’ attendance and completion rates. Due to security concerns, many parents are reluctant to send their daughters to remote government schools [6]. In addition, the risk of aggressive attacks toward enrolled girls has led to schools being secured and declared “zones of peace and neutrality” [7]. Considering recent changes in the Afghan government, as the Taliban are back in power, there are reasons to fear further disruption in access to education, especially for girls.

Attending school has been shown to mitigate mental health risks among children. For instance, in one study conducted in large cities in China where migrant children had limited access to school, attending public school served as a protective factor for child mental health, including externalizing problems and peer relations, as compared to migrant children attending their own private schools, located in a poor environement where teaching is following low standards. In addition, the protective effect of public school attendance was even more salient among girls than boys, and for younger children compared to older children [8]. More recently, during the SARS-coV-2 pandemic, school closures have been associated with a deterioration of child mental health [8]. Moreover, the mental health deterioration was largest in low income children [9].

Afghan mental health has been one of the Ministry of Public Health’s priorities, and became a part of the Basic Package of Health Service in 2003. A national mental health strategy was developed for 5 years (2010–2014) and revised in 2015. The biggest strength of this strategy was the integration of mental health services into each level of the healthcare system. In 2017, a national mental health survey was proposed to evaluate the [10] state of mental health of the population adult and children and their access to care for these problems [11, 12].

*The present study concerns the assessment of child mental health in war-torn Afghanistan*. To achieve this goal, we conducted the first nationally-representative survey to examine exposure to terrorist threat and school attendance on child mental health. The objectives of the study were to determine (1) the association between levels of terrorist threat and child mental health problems, and (2) whether attending school serves as a protective factor for child mental health.

## Methods

### Sample and procedures

Trained staff members administered a household survey in each of these eight regions of Afghanistan: (1) Eastern; (2) South Eastern; (3) Southern; (4) Western; (5) North Western; (6) North Eastern; (7) Central Kabul; (8) Central Bamiyan. A multi-stage stratified cluster sampling recruitment method was used. First, two provinces in each region (n = 16) were randomly selected for participation, representing almost half of the total number of provinces in the country (n = 34). Within each province, the Central Statistical Organization (CSO) randomly sampled clusters, resulting in a total of 320 clusters countrywide. For security reasons, approximately 10 clusters were replaced with a nearby cluster in the same district or nearby district. Surveyors completed questionnaires for 14 households randomly selected within each cluster. Recruitment goals for each region were 542 adults, for a total of 4336 adults countrywide. Household participation rate was, on average, 90% (ranging from 86 to 93% depending on the region). Data were collected between January and July 2017.

Local staff members received a 5-day training that provided instructions on survey method, cluster identification, a comprehensive and detailed review of questionnaires, and one day in the field testing the questionnaires. In total, the data collection process mobilized 64 teams of two individuals (one male and one female staff). There was one supervisor for each province to manage the administrative aspects and coordinate the data collection process. Interviews were administered either in Dari or Pashtu according to the language spoken in the selected household. Language/ethnic specificities in the different provinces were taken into consideration by recruiting interviewers fluent in either language. Questionnaires were read aloud to participants.

In every household with at least one child aged 4 to 15 years old, one index child was randomly selected and their parent was invited to complete a separate survey on the psychosocial wellbeing of that child. Overall, 4435 parents were given the offer to complete the questionnaire regarding their child, and 3784 (85.32%) completed it. In the present study, considering our focus on school enrollment and that school starts at age 7, we further restricted the sample to the parents who reported on an index child that was 7 years old or more, providing a final sample of 2707 responses. Among them, respondents were primarily female (73.96%): 63.87% were the mother (plus 0.41% who were the stepmother), and 18.54% were the father; the rest of the responses were completed by another member of the family (6.43%, in equal gender proportion) or a foster parent (9.75%), mostly women.

### Measures

**Fig. 1:**
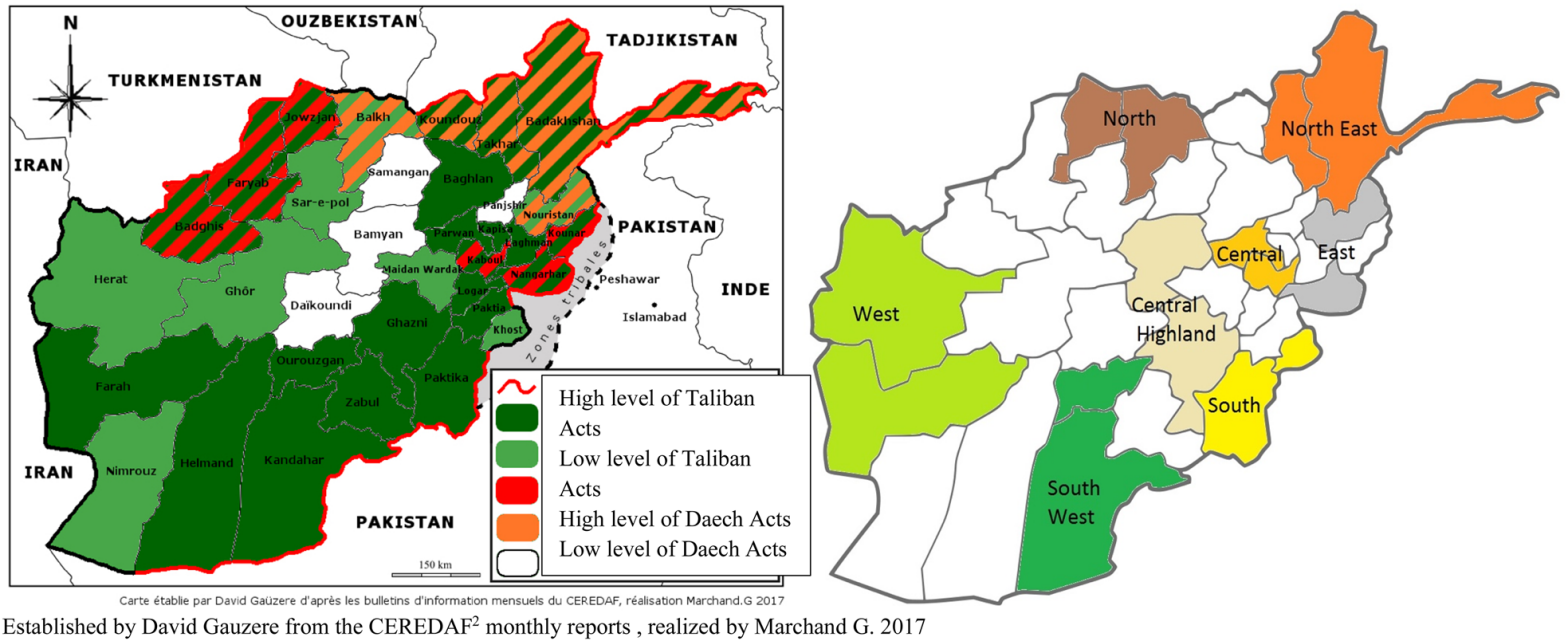
Regional map of Afghanistan: on the top Levels of danger, on the bottom randomized region (Colors indicated the regions that have been selected; the remaining regions are in white). Established by David Gauzere from the CEREDAF (http://ceredaf.free.fr/) monthly reports, realized by Marchand G. 2017

Level of terrorist threat: To estimate exposure to dangerous situations, each of the 16 provinces was assigned a score in accordance with levels of Taliban or Isis (Daesh) activities reported by a French NGO “Centre d’Etudes et de Recherches Documentaires sur l’Afghanistan” (CEREDAF) for the year 2017 (see Fig. 1). These scores: 0 “absent”, 1 “moderate”, 2 “high”, provided for each terrorist organisation, were combined in order to obtain a global risk score such as “very high” when both activities reach high level, “high” when one of the risks is high, and “low/medium” when one or both risks were moderate or low.

Sociodemographic characteristics: Gender was recorded as a binary variable (male or female) for the parent and for the index child. Age was recorded as a continuous variable. As 74.75% of the parents never attended school and an additional 3.17% did not complete primary school, parental level of education was dichotomised into less than primary school (76.28%) versus primary school or more (23.72%). Ethnicity was reported as a categorical variable with the following options: Tajik, Pashtun, Uzbek, Hazara, and “other”. Urbanicity of place of residence was measured using a binary categorical variable (rural vs urban). *In addition to the parent questionnaire, a household questionnaire allowed for a self-declared evaluation of the economic situation of the family from very poor to very rich, the total number of persons living in the household, and the number of alive children of the household respondent.*

School attendance: Parents were asked to indicate whether or not their child currently attended school.

Parent-reported child mental health: Child mental health was assessed with the parent version of the Strengths and Difficulties Questionnaire (SDQ) [13]. The SDQ contains 25 questions. Each item is scored as ‘not true’, ‘somewhat true’ or ‘certainly true’ over the past 6 months. The questionnaire is divided into five subscales of five items each: “Hyperactivity/Inattention”, “Emotional problems”, “Conduct problems”, “Peer problems” and “Prosocial behaviors”. In addition, the complete SDQ version assesses the level of impairment in four different areas of the child’s daily life. For each of four SDQ dimensions (Emotional, Conduct, Hyperactivity-Inattention and Peer relationship problems) plus “Total Difficulties” (the sum of the four scales’ scores), cut points corresponding to “normal”, “borderline” and “abnormal” have been provided by its author, who also provided “impairment” cut points with the same proposed categories [13, 14]. For each dimension, we have presented the results for the “abnormal” category, and pooled “borderline” and “normal” categories, in order to obtain a binary variable indicating presence/absence of the probable problems in each domain. *In addition, we have recalculated the results using “normal” versus pooled “borderline and abnormal” as a sensitivity analysis.*

The SDQ had been translated and validated in Dari and Pashtu and have been previously used in Afghanistan [15]. Moreover, in the present sample, the instrument yielded a Cronbach’s alpha for the total scale of 0.84; 0.70 for the emotional scale, 0.54 for conduct scale 0.60 for hyperactivity/inattention, 0.42 for peer relations, 0.65 for prosocial. As expected, the total difficulties scores were correlated with each subscales (0.78, 0.68, 0.73, 0.55), with the exception of the prosocial scale, which does not assess mental health problems. This pattern was quite similar to what was obtained in a similar Dutch study [16].

### Data analysis

For each of four main SDQ dimensions (SDQ Emotional, SDQ Conduct, SDQ Hyperactivity-Inattention and SDQ Peer relationship problems) plus “Total Difficulties” (the sum of these four scales’ scores), cut points corresponding to “normal”, “borderline” and “abnormal” have been provided by its author, who also provided “impairment” cut points with the same proposed categories [13, 14]. For each such dimension (6 in total), we have presented the results for the “abnormal” category, having pooled together “borderline” and “normal” categories, in order to obtain a binary variable presence/absence of the abnormal score. This dichotomization has been used for monovariate and multivariate analyses.

One-way prevalence comparisons used Pearson chi-square tests; multivariate logistic regressions used the STATA “logit” command. Multivariate models were estimated for each of the MH dimensions; in order to evaluate the specific effect of school attendance, we controlled for the other main potential determinants: child and parent sociodemographic characteristics, region of residence, urbanicity and level of terrorist threat. In addition, an interaction term was calculated for gender and school attendance, controlling for the same determinants. AOR (Adjusted Odd Ratios) were presented with their 95% confidence intervals. Analyses were performed using Stata/IC 15.1 [17].

## Results

The average number of people living in a household was 9.51 (SD = 0.86), and was higher in rural areas than in urban areas (9.77 versus 8.77, p < 0.0001); differences across regions were significant as well. The average number of children alive from the household respondent was 5.39 (SD = 0.43). Each parent and child sociodemographic characteristic varied significantly by region of residence, as did school attendance, ranging from 60.15% to 91.93% (Table 1). In addition, one third of the sample was exposed to a high level of terrorist threat (31.55%) while another third was exposed to a very high level of threat (36.17%).

**Table 1:** Sample characteristics by geographic region of residence within Afghanistan (%)

	Total sample	Region of residence within Afghanistan								Between-region comparisons	
		Kabul /Central	South	East	South West	West	North	Central Highland	North East		
	n = 2,707	n = 322	n = 376	n = 342	n = 404	n = 316	n = 300	n = 310	n = 337	Chi^2^	p-value
**Level of terrorist threat**											
Low to moderate	32.29	0	59.04	7.02	0	85.76	84.67	33.23	0	3.6e+03	< 0.0001
High	31.55	13.66	40.96	0	100	14.24	0	66.77	0		
Very high	36.17	86.34	0	92.98	0	0	15.33	0	100		
**Environment **											
Rural	73.29	23.91	93.88	88.01	62.62	72.15	63.33	93.55	86.65	654.962	< 0.0001
**Parent characteristics**											
Gender											
Male	26.04	25.47	21.54	33.04	30.45	22.15	17.67	24.84	31.45	35.5390	< 0.0001
Female	73.96	74.53	78.46	66.96	69.55	77.85	82.33	75.16	68.55		
Ethnicity											
Tajik	26.82	56.21	1.06	2.92	0.99	51.58	44.33	1.00	59.35	3.4e+03	< 0.0001
Pashtun	50.79	27.02	98.94	87.43	95.30	37.97	13.00	19.03	4.15		
Hazara	10.64	12.11	.00	.00	3.47	5.38	3.00	67.10	0.30		
Uzbek	6.21	0.31	.00	.00	0.25	.00	15.67	.00	35.31		
Other	5.54	4.35	.00	9.65	.00	5.06	24.00	3.87	0.89		
Education level											
Low	76.28	66.15	80.59	80.12	81.68	74.05	77.67	70.32	77.15	38.8313	< 0.0001
Moderate to High	23.72	33.85	19.41	19.88	18.32	25.95	22.33	29.68	22.85		
Economic situation											
Very poor	6.19	4.09	12.63	11.31	4.98	5.13	4.76	1.32	3.96	232.2526	< 0.0001
Poor	33.85	24.53	32.53	38.39	30.10	35.90	40.48	31.35	38.72		
Middle income level	55.65	71.38	45.97	48.81	52.74	58.65	54.76	60.40	55.49		
Rich/very rich	4.32	0	8.87	1.49	12.19	0.32	0	6.93	1.83		
**Index child characteristics**											
Gender											
Male	53.16	49.07	58.51	62.57	55.69	48.42	45.67	49.68	52.82	30.8438	< 0.0001
Female	46.84	50.93	41.49	37.43	44.31	51.58	54.33	50.32	47.18		
Age											
7–9 years	42.87	41.61	45.99	51.91	39.36	38.85	40.47	47.42	37.39	38.0463	0.001
10–12 years	39.76	38.82	37.43	37.54	38.37	44.27	39.80	39.03	43.62		
13–14 years	17.36	19.57	16.58	10.56	22.28	16.88	19.73	13.55	18.99		
School attendence rate (both genders)											
No	21.91	8.07	21.54	25.44	39.85	20.25	19.67	19.35	16.32	123.2934	< 0.0001
Yes	78.09	91.93	78.46	74.56	60.15	79.75	80.33	80.65	83.68		
School attendence rate for girls	70.58	88.41	61.54	54.69	50.84	74.23	79.14	71.15	83.02	99.1110	< 0.0001
**Number of people in the household**	9.51	8.26	13.50	12.47	10.23	7.46	7.69	8.45	7.89	131.16	< 0.0001
Number of household respondent’s children who are alive	5.39	5.11	6.37	6.20	5.64	5.10	4.86	4.91	4.94	25.71	< 0.0001

**Fig. 2:**
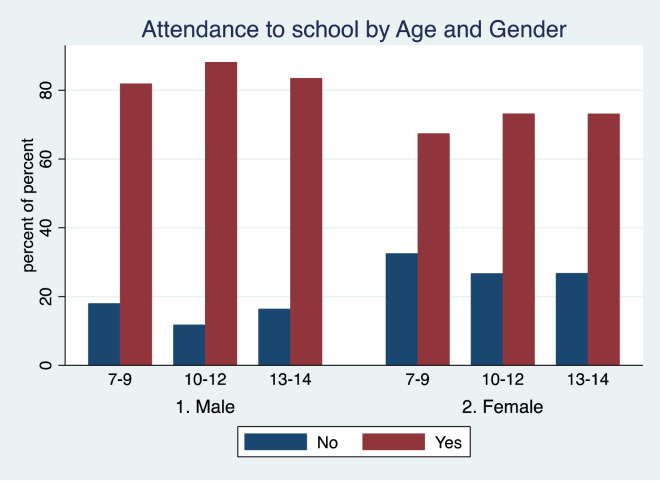
Attendance to school by Age and Gender: Yes = Attendance, No = No-attendance

A total of 29.42% of girls did not attend school vs 15.29% of boys (P < 0.001) (Fig. 2). This was true in primary school (29.89% of girls vs 14.94% of boys, P < 0.001) as well as in secondary education (27.15% of girls vs 16.93% of boys, P < 0.01). This trend persisted across all age groups.

In addition to the child’s age, gender, and region, several other factors were associated with school attendance (Table 2), including urbanicity, with those in a rural area significantly less likely to attend school (P < 0.001). Parental education was particularly significant, as children with a parent with very little or no education were much more likely to not attend school (24.50%) compared to those with a parent with at least some education (13.55%), P < 0.001. Each of these factors remained significant in multiple regression analyses.

**Table 2:** Determinants for school attendance (logistic)

	OR	95% CI		P
**Children**				
Age (by year)	1.08	1.03	1.13	**0.002**
Gender (girl vs boy)	0.37	0.30	0.45	**< 0.0001**
**Parents**				
Economic situation				
Very poor (ref)				
Poor	1.37	0.93	2.03	0.112
Middle income level	2.05	1.39	3.01	**< 0.0001**
Rich	2.46	1.36	4.43	**0.003**
Education				
Mod-high vs low	1.65	1.26	2.17	**< 0.0001**
Ethnicity				
Tajik (ref)				
Pashtun	0.63	0.43	0.92	**0.016**
Hazara	1.88	1.05	3.38	**0.035**
Uzbek	0.82	0.49	1.37	0.448
Other	0.86	0.52	1.44	0.565
**Environmen**t				
Rural vs urban	0.32	0.24	0.44	**< 0.0001**
Danger level				
Low/mild (ref)				
High	0.49	0.34	0.70	**< 0.0001**
Very high	1.25	0.70	2.23	0.455
Region				
Kabul (ref)				
South	1.23	0.61	2.51	0.561
East	0.53	0.31	0.93	**0.027**
South West	0.44	0.22	0.89	**0.022**
West	0.77	0.39	1.52	0.443
North	0.55	0.29	1.05	0.071
Central High Land	0.82	0.39	1.73	0.600
North East	0.63	0.34	1.17	0.143

### Parent-reported child mental health problems by regional and parental characteristics

In the country as a whole, mental health problems, as evaluated by SDQ scales, were common, with half of the children having an elevated total difficulties score (52.75%). Peer relationships problems were reported among 82.86% of children. The association between level of terrorist threat and mental health problems differed by type of mental health problem. The prevalence of emotional, conduct problems and total difficulties increased as the level of terrorist threat increased.

There were no differences between rural and urban areas, except for any impairment, which was greater in rural than in urban areas: 13.46% versus 9.42% (P = 0.005). Several mental health dimensions were associated with parental education, including total difficulties (55.30% for children with parents without any education vs 44.55% among children with parents with some education, P < 0.0001), emotional problems (41.79% vs 30.84%, P < 0.0001) and hyperactivity/inattention problems (16.27% vs 12.46%, P = 0.019) (Table 3). *Changing the SDQ case definition (0 versus 1 or 2) increased prevalence as expected, but did not change the differences between those who attend and did not attend schools.*

**Table 3:** Parent-reported child mental health problems in the prior 6 months by regional and parental characteristics (%)

SDQ dimensions	Hyperactivity/inattention	Conduct	Emotional	Peer relation	Total difficulties	Any Impairment
Total sample	15.37	51.98	39.19	82.86	52.75	12.38
	30.37	72.18	52.35	93.35	72.40	15.53
**Level of terrorist threat**						
Low to moderate	14.76	**50.34**	**32.84**	81.58	**43.14**	13.96
High	14.75	**50.00**	**43.09**	82.44	**58.08**	10.32
Very high	16.45	**55.16**	**41.47**	84.37	**56.69**	12.78
**Parent characteristics**						
Gender						
Male	15.91	**56.64**	38.78	83.04	54.13	12.38
Female	14.75	**46.69**	39.67	82.65	51.18	12.39
Ethnicity						
Tajik	**12.26**	**57.85**	40.22	83.61	**53.17**	**9.92**
Pashtun	**16.87**	**49.67**	38.76	82.98	**54.84**	**16.30**
Hazara	**15.97**	**51.04**	36.11	78.82	**44.79**	**3.48**
Uzbek	**19.05**	**57.75**	44.64	86.90	**60.12**	**4.17**
Other	**11.33**	**40.00**	38.00	81.33	**38.67**	**14.67**
Education level						
Low	**16.27**	52.69	**41.79**	83.10	**55.30**	12.89
Moderate to high	**12.46**	49.69	**30.84**	82.09	**44.55**	10.75
Economic situation						
Very poor	16.36	**51.52**	**46.06**	78.18	57.58	**25.00**
Poor	13.53	**56.21**	**42.68**	82.71	54.21	**13.75**
Average	16.12	**50.03**	**36.82**	83.48	51.05	**9.78**
Rich and very rich	18.26	**46.09**	**34.78**	82.61	54.78	**18.26**

Bold signifies significant differences using Pearson chi square tests at p < 0.05

### Parent-reported child mental health problems by child characteristics and school attendance

Conduct problems were more frequent among boys than among girls (P < 0.0001) (Table 3). No other gender differences nor age differences were observed. Those who attended school were less likely to have emotional problems, and impairment. In contrast, children who attended school were more likely to exhibit peer relationship problems. Externalizing problems such as hyperactivity/inattention or conduct problems, did not significantly differ based on school attendance. (Table 4).

**Table 4:** Parent-reported child mental health problems by child characteristics and school attendance

Child characteristics	Hyperactivity/inattention problems	Conduct problems	Emotional problems	Peer relationship problems	Total difficulties	Any impairment
	%	%	%	%	%	%
**Gender**						
Male	15.91	**56.64**	38.78	83.04	54.13	12.38
Female	14.75	**46.69**	39.67	82.65	51.18	12.39
**Age**						
7–9 years	14.85	52.50	39.98	81.43	53.28	12.19
10–12 years	14.99	52.42	39.01	83.89	53.07	11.82
13–14 years	17.70	49.68	37.95	84.22	50.75	14.32
**School attendence**						
0/1 versus 2 No	14.84	51.77	**48.06**	**75.89**	55.99	**16.07**
0/1 versus 2 Yes	15.52	52.03	**36.71**	**84.82**	51.84	**11.35**
0 versus 1 /2 No	31.37	69.65	**62.73**	**88.53**	72.18	**18.61**
0 versus 1 /2 Yes	30.09	72.89	**49.43**	**94.70**	72.47	**14.66**

Bold signifies significant differences using Pearson chi square tests at p < .05

### Multivariate associations between level of terrorist threat, parental, and child characteristics and school attendance with child mental health problems

In a multiple logistic regression, controlling for all relevant regional, parental and child characteristic, the level of terrorist threat was significantly associated with increased odds of each of the mental health dimensions considered, with the exception of impairment (Table 5).

**Table 5:** Multivariate associations between level of terrorist threat, parental, and child characteristics and school attendance with child mental health problems

	Emotional			Conduct			ADHD			Peer relations			Total difficulties			Impairement		
	OR	95CI		OR	95CI		OR	95CI		OR	95CI		OR	95CI		OR	95CI	
**Children**																		
Age (by year)	0.99	0.96	1.03	0.98	0.95	1.02	**1.05**	1.00	1.10	*1.04*	1.00	1.09	0.99	0.95	1.02	**1.06**	1.00	1.12
Sex (girl vs boy)	0.95	0.80	1.12	**0.61**	0.52	0.72	0.91	0.73	1.14	1.09	0.88	1.35	**0.83**	0.70	0.98	1.02	0.79	1.31
School attendance	**0.67**	0.54	0.82	0.91	0.74	1.12	1.06	0.80	1.41	**1.93**	1.50	2.47	0.93	0.76	1.15	**0.70**	0.53	0.94
**Parents**																		
Economic situation																		
Very poor (ref)																		
Poor	0.86	0.61	1.21	1.11	0.79	1.56	0.86	0.54	1.38	*1.45*	0.95	2.21	0.90	0.63	1.28	**0.60**	0.39	0.90
Middle income level	**0.69**	0.49	0.97	0.83	0.59	1.16	1.09	0.69	1.71	*1.48*	0.98	2.23	0.76	0.54	1.07	**0.42**	0.28	0.64
Rich	*0.63*	0.38	1.04	0.80	0.48	1.31	1.33	0.69	2.55	1.38	0.73	2.59	0.90	0.54	1.49	0.86	0.46	1.61
Education																		
Mod-high (vs low)	0.75	0.61	0.93	1.03	0.84	1.25	0.84	0.63	1.13	1.03	0.79	1.34	*0.83*	0.68	1.02	1.06	0.77	1.46
Sex (Woman vs Man)	**1.57**	1.28	1.92	**1.66**	1.37	2.02	**1.76**	1.31	2.38	**1.62**	1.27	2.07	**2.36**	1.93	2.87	**1.56**	1.13	2.15
Ethnicity																		
Tajik (ref)																		
Pashtun	0.83	0.62	1.11	0.86	0.65	1.14	1.36	0.92	2.02	**0.67**	0.46	0.96	0.86	0.64	1.14	0.97	0.64	1.48
Hazara	**0.66**	0.44	0.98	0.71	0.49	1.04	1.30	0.77	2.20	**0.63**	0.39	1.01	**0.63**	0.43	0.92	**0.21**	0.09	0.47
Uzbek	0.91	0.62	1.34	0.86	0.58	1.28	1.19	0.70	2.02	1.33	0.77	2.29	1.13	0.76	1.67	0.73	0.30	1.75
Other	1.08	0.72	1.61	**0.64**	0.43	0.96	0.69	0.37	1.27	0.91	0.55	1.53	*0.70*	0.47	1.05	1.13	0.63	2.05
**Environment**																		
Rural (vs urban)	1.00	0.81	1.25	0.97	0.78	1.20	0.96	0.71	1.29	1.07	0.80	1.42	0.91	0.73	1.13	*1.37*	0.97	1.94
Danger level																		
Low/mild (ref)																		
High	**1.62**	1.22	2.16	0.98	0.74	1.30	1.16	0.79	1.69	**1.40**	0.97	2.02	**1.99**	1.49	2.65	*0.68*	0.45	1.03
Very high	**2.01**	1.28	3.17	**2.43**	1.53	3.87	**7.79**	3.99	15.20	**3.61**	2.01	6.51	**3.87**	2.41	6.22	0.96	0.48	1.93
Region																		
Kabul (ref)																		
South	*1.69*	0.96	2.97	1.63	0.93	2.87	**9.16**	3.94	21.30	**4.67**	2.32	9.41	**2.42**	1.37	4.29	1.31	0.56	3.05
East	0.87	0.59	1.28	0.56	0.38	0.82	1.18	0.70	1.98	**1.74**	1.06	2.85	0.87	0.60	1.27	1.46	0.86	2.48
South West	1.37	0.78	2.40	1.88	1.07	3.29	**5.25**	2.15	12.79	**4.29**	2.19	8.41	**2.35**	1.33	4.14	0.68	0.28	1.65
West	1.31	0.78	2.23	2.48	1.46	4.20	**6.58**	2.89	14.95	**3.59**	1.88	6.87	**2.59**	1.52	4.41	0.82	0.37	1.82
North	1.35	0.84	2.18	1.37	0.84	2.24	**9.41**	4.66	19.01	**2.72**	1.46	5.04	**1.66**	1.01	2.73	**0.35**	0.16	0.77
Central High Land	**2.13**	1.20	3.78	**2.53**	1.42	4.48	**7.63**	3.11	18.74	**2.89**	1.46	5.70	**2.27**	1.27	4.04	1.13	0.46	2.78
North East	*1.47*	1.00	2.17	1.19	0.80	1.75	0.96	0.55	1.69	0.79	0.47	1.32	0.96	0.65	1.42	**0.27**	0.13	0.56

Logistic regression on the presence/absence of above SDQ scales cut; controlled for level of danger, place of residence (rural /urban), ethnicity, child age (continuous), gender and attendance to school (yes/no), parent education (no education versus some), economic situation, gender and region

Bold signifies p<0.05; Italic p<0.10

In the same model, attending school was associated with lower odds of emotional problems (AOR = 0.65, P < 0.0001) and lower odds of impairment (AOR = 0.67, P = 0.007). In contrast, school attendance was associated with greater odds of peer relationship problems (AOR = 1.96, P < 0.001). Conduct and hyperactivity/inattention scores did not seem to differ by school attendance status. *Changing the SDQ case definition (0 versus 1 or 2) provided identical results.*

## Discussion

In Afghanistan, a country with a long history of war-related violence, the level of terrorist threat was found to be significantly associated with child mental health. Within this high-risk sample, the level of exposure to terrorist threat played a significant role. Children living in areas with high levels of terrorist threat were more likely to have emotional as well as conduct problems, hyperactivity/inattention, and peer relationships problems, as compared to children living in areas with lower levels of threat. Children living in war zones are significantly more likely to be exposed to traumatic life events, as has been documented in Kabul [3] and among war refugees [18].

In addition, child mental health varied by region, independently of the level of violence. Children in the Kabul area were at greater risk for emotional problems compared to most other regions. This replicates findings from another study conducted in Afghanistan in which children living in the North (Mazar) and Northeast had lower prevalence of impairment due to mental health problems [15]. In that study, the difference was related to the multiplicity of current social and economic stressors in the capital, where overcrowding, high living costs, widening inequalities, pressure on resources, and day-to-day stressors might compound other adversities directly related to war [19]. In contrast, peer relationship and SDQ total difficulties were higher in most other regions compared to the Kabul area. This may be due to stronger discipline and traditional education in these regions. Curiously, the ethnicity effect has not been evaluated in the previous Afghan studies; in the Kabul study, 90% of the sample were Hazara [3] and the second study did not address ethnic composition. In our sample, Hazara children had fewer problems in nearly each category, except emotional, than the Tajik; Pashtun had fewer peer relationship problems than Tajik. Ethnic differences have often been studied in child mental health surveys and correspond to a large scope of behavioral differences which concern child education and expectation toward school achievement as well as discipline, strict obedience and corporal punishment [20, 21], which in turn influence children’s mental health and their parental relationships.

Overall, in the present study, Afghan children were identified as presenting particularly high levels of mental health problems. Over one half (52.75%) of the children had an above-threshold score on the total difficulties scale. These figures should be compared to prior research on school children in Europe that reported 19% of children meeting that threshold in Lithuania, 14.4% in Turkey, 13% in Bulgaria and Romania, 9.5% in Germany, 7% in the Netherlands and 3.4% in Italy [22]. Furthermore, in a sample of Arab children in the Gaza strip, the prevalence of above threshold children was estimated at 16.3% among 11-year-olds [23]. The only reports of significantly higher proportions of children presenting with above-threshold scores were found in one primary school-based survey in Karachi with a prevalence at 31% [24]. Yet, none of these other rates come close to the rates reported here.

School attendance was associated with lower odds of emotional problems and impairment for boys and for girls. This is a particularly important finding and had never previously been documented. Indeed, all prior child mental health surveys had been conducted in schools, ignoring, by design, those who did not attend. There are major implications regarding this finding, especially in light of the return of the Taliban, which places school access at increased risk for millions of children, mostly girls.

In contrast, attending school was a risk factor for peer relations problems, as violence at school between school children, as well as between teachers and schoolchildren, has been reported as very frequent in Afghanistan [25]. This finding may have a cultural component to it, whereby Afghan parents have been reported to take pride in some children’s aggressive behaviors, including against girls, as such behaviors may reflect combativeness and patriarchism which is valued in that culture, and therefore may have been readily reported by parents [26]. However, harassment has been documented as detrimental for mental health [27]. Programs have been developed and successfully implemented in Afghanistan [28] to decrease the level of violence both at schools and at home. The evaluation of the latter program suggested that conducting what they called “peace education” with children in schools, and with adults in the community, may lead to a reduction in various forms of violence, including children’s peer violence, corporal punishment of children both at school and at home, and reports of children witnessing domestic violence against women at home.

Contrary to what has been described in most Westernised surveys, there were no gender differences for emotional and ADHD, as well for SDQ peer relationships, impact and total difficulties; a lack of gender difference has already been reported in a study on school children in Karachi for SDQ emotional and peer relationship [24]; in addition, an unexpected high level of emotional disorders among boys has been reported in a previous study on the same country [15].

However, the gender of the respondent was highly significant, as women consistently reported higher levels of any type of problems. This was reported in the previous Afghan study [15] and was attributed to the poorer mental health of women as compared to men, leading potentially to a more negative evaluation of their children’s mental health, which potentially emphasizes negative problems. It must be added that Afghan men may have difficulties admitting problems in front of the interviewer, at least for themselves, [29] whereas this may not be present for women, a trend that may apply to their children’s mental health.

## Limitations

There are several limitations to this study. First, this is a cross-sectional study, and as such prevents any causal associations between study variables. Second, while the SDQ has been used in different cultural settings, including Afghanistan, and validated for use in the languages used here, its scoring may not apply uniformly across cultures [30, 31]. Chronbach’s alpha for SDQ scales, although high globally, is rather low for peer relations in Afghanistan, as was found in other studies. Third, child mental health was solely reported by parents, which may have led us to underestimate the prevalence of emotional problems. That being said, research has found concordance between parental evaluation of child mental health and clinical assessment [32] and parents and teachers are relatively concordant [33]. This does not preclude some discrepancies between child- and parent-report [34]. Fourth, parental mental health was not assessed, which is an important child mental health determinant. Fifth, there is also the potential for sampling bias. Although participation response rate was high, some highly dangerous areas of the country were excluded from the study, and the classification of terrorist threat levels was based on regional number of attacks that may vary inside the same region. However, we have established in another part of the sample that to live in a very highly dangerous area is a main predictor of individual trauma [35]. Lastly, the proportion of girls differed across regions, which may reflect some reticence among the most conservative parents not to take part in the survey.

## Conclusions

Afghan children living in dangerous areas are suffering from a high level of traumatic events reflected by high levels of symptoms: more than half of them have high difficulties scores, which are much higher than any other countries reporting child mental health symptoms. *Adequate interventions, including psychosocial ones should be developed into the primary care as planed in the Afghanistan Mental health Strategy.*

This concerns internalised as well as externalized symptoms, including conduct problems and peer relationships, which are particularly elevated. *Since violence between students has proved to be amenable to prevention programs, as has violence between parents and children, these prevention programs should be promoted at the country level.*

In a highly exposed child population, attending school is protective against some mental health problems for boys as well as for girls, despite a certain level of violence at school. This work illustrates that good quality epidemiological data could, and should, be collected in hard to reach areas, especially in areas where children are exposed to traumas. Research on protective factors needs to be conducted in places where children’s lives, especially the lives of young girls, are far from what is experienced in many other places in the world. How the protective role of attending school could be maximized for both boys and girls is a critical topic of future research. *In the mean time, children access to school should be secure for boys and girls all around the country and training of female teachers prioritised.*

Lastly, epidemiological studies must continue on the children leaving in Afghanistan, as they are facing many additional adversities [36], as well as children who have to leave the area, with or without their parents.

## Data Availability

The datasets generated and/or analysed during the current study are not publicly available due to the mandatory request of authorisations from the funder and the Afghan ministry of public health but are available from the corresponding author on reasonable request.

## Footnotes

1 Gross Enrolment Ratio (GER) is a statistical measure used in the education sector, to determine the number of students enrolled in school at several different grade levels (like elementary, middle school and high school), and use it to show the ratio of the number of students who live in that country to those who qualify for the particular grade level.
